# Complete chloroplast genome of a rare deciduous tree species, *Sinomanglietia glauca* (Magnoliaceae)

**DOI:** 10.1080/23802359.2019.1668730

**Published:** 2019-09-23

**Authors:** Jiang-Ping Li, Ling Cui, Li-Hong Qiu, Chun-Ce Guo, Guang-Yao Yang, Wen-Gen Zhang

**Affiliations:** College of Forestry, Jiangxi Agricultural University, Nanchang, P.R. China

**Keywords:** Magnoliaceae, *Manglietia decidua*, phylogeny

## Abstract

The complete chloroplast genome of a rare deciduous tree species with ornamental value, *Sinomanglietia glauca*, was first determined. It was 160,170 bp in length, including a pair of inverted repeat (IR, 26,567 bp) regions separated by a small single copy (SSC, 18,842 bp) sequence and a large single copy (LSC, 88,194 bp) sequence. The chloroplast genome contained 132 genes, consisting of 87 CDS, 8 rRNA genes, and 37 tRNA genes. Thirty-four SSR sites were detected in the chloroplast genome. The phylogenetic analysis revealed that all sampled *Manglietia* species were clustered together, and *S. glauca* was placed as sister to the clade *Manglietia*, indicated that the genus *Sinomanglietia* may be legitimate and should be recovered.

*Sinomanglietia glauca* (Yu (1994, p. 203) is a rare deciduous tree species of Magnoliaceae with ornamental value and has been listed as the category I of the National Key Protected Wild Plants (NKPWP) in China (under a synonym of *Manglietia decidua* Q. Y. Zheng). Discretely limited to partial regions of Jiangxi and Hunan, *S. glauca* is distributed below 1000 m along hillsides and in the understory of evergreen and deciduous broad-leaved forest (Yu 1999; Hou et al. 2006). However, its taxonomic status is hitherto controversial (Zheng 1995; Azuma et al. 2001; Lin and Yu 2004; Kumar 2006). Here, we determined the complete chloroplast genome sequence of *S. glauca* and re-evaluated its phylogeny.

Sequencing materials were collected from the arboretum of Jiangxi Agricultural University, China (28°45′40″N, 115°49′31″E), and Voucher specimens were deposited in the herbarium of the College of Forestry, Jiangxi Agricultural University, China with the accession number Li and Cui 0421 (JXAU!). Approximately, 5.3 Gb of clean reads data was obtained, and contigs were assembled by SPAdes 3.13.0 (Bankevich et al. 2012). Using Geneious 9.0.5 (Kearse et al. 2012), *de novo* and reference-guided methods were combined to assemble a chloroplast genome. Then, the webserver DOGMA (Wyman et al. 2004) was applied to annotate the complete chloroplast genome and simple sequence repeats (SSR) were detected by MISA (http://pgrc.ipk-gatersleben.de/misa). The complete chloroplast genome of *S. glauca* was 160,170 bp in length and showed a typical quadripartite structure including a pair of inverted repeat regions (IR: 26,567 bp), a large single-copy region (LSC: 88,194 bp) and a small single copy region (SSC: 18,842 bp). The GC content in the chloroplast genome of *S. glauca* is 39.3% and the corresponding values in LSC, SSC, and IR regions are 38.0, 34.3, and 43.2%, respectively. The complete chloroplast genome contained 132 genes, including 87 protein-coding genes, 8 rRNA genes, and 37 tRNA genes. Twenty-four genes were present in the IR region and 34 SSR sites were detected in the chloroplast genome of *S. glauca*.

To re-evaluate the phylogenetic status of *S. glauca*, an additional 29 complete chloroplast genomes within the family Magnoliaceae (Xia et al. 2008), together with five species as outgroup, were downloaded from NCBI. All sequences were aligned with MAFFT 7.409 (Katoh and Toh 2010), and then manually adjusted using GeneDoc 2.7 (Nicholas 1997). The phylogenetic tree was calculated with Bayesian method using MrBayes 3.2.7 (Ronquist et al. 2012). The phylogenetic analysis reveals that all sampled *Manglietia* species were clustered together with high support value, and *S. glauca* was placed as sister to the clade *Manglietia* (Figure 1), which indicated that the genus *Sinomanglietia* may be legitimate and should be recovered. Meanwhile, *Manglietia fordiana* var. *calcarea* (MF990562.1) was not grouped with *M. fordiana* (MH394398.1), indicating that the former may be an independent species, which partly supports recent revisions ‘renamed as *Manglietia aromatica* var. *calcarea* (X. H. Song) Sima et S. G. Lu’ (Sima et al. 2014). Furthermore, the chloroplast resource still will provide molecular genetic information for DNA barcoding, conservation genetics, and breeding of *S. glauca* in the future.

**Figure 1.**
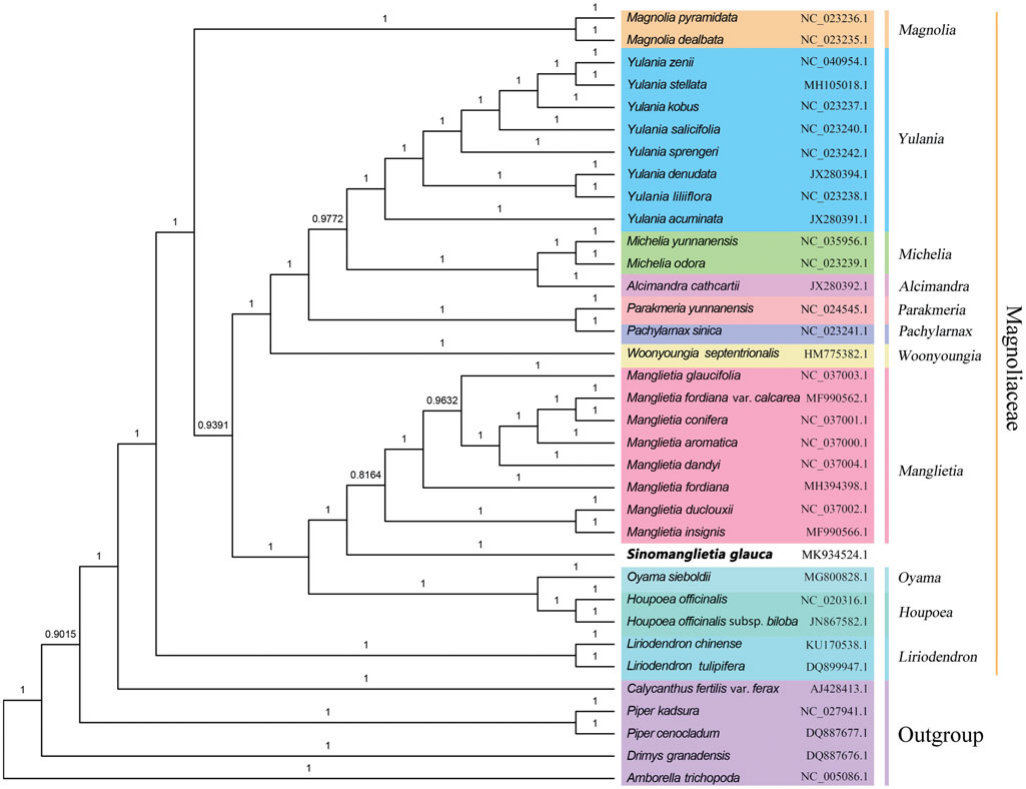
Phylogenetic tree using Mrbayes 3.2.7 based on 35 chloroplast genomes of Magnoliaceae and otherrelative species with corresponding GenBank accession number.
